# Cervical Neutrophil Infiltration in Relation to Macroscopic Cervix Appearance, Cytological Endometritis, and Reproductive Stage in Postpartum Grazing Dairy Cows

**DOI:** 10.3390/ani16213343

**Published:** 2026-10-23

**Authors:** Darío Vallejo-Timarán, Andrés Timarán-Rivera, Eliana Revelo-Gómez, José Escobar-Riomalo, Juan Astaiza-Martínez, John Montoya-Zuluaga, Viviana Castillo-Vanegas, Rejean Lefebvre

**Affiliations:** 1 Obonuco Research and Technology Transfer Center, Corporación Colombiana de Investigación Agropecuaria—AGROSAVIA, Km 5 Vía Pasto—Obonuco, Pasto 520038, Colombia; etimaran@agrosavia.co (A.T.-R.); erevelo@agrosavia.co (E.R.-G.); 2 Master’s Program in Agricultural Sciences, Center for Research and Postgraduate Studies in Agricultural Sciences, University of Nariño, Las Acacias Vipri, Cra. 33 #5-121, Pasto 520001, Colombia; 3 Research Group GISCA, Faculty of Veterinary Medicine and Zootechnics, Institución Universitaria Visión de las Américas, Calle 34A #76-35, Laureles, Medellín 050031, Colombia; jose.escobar@uam.edu.co (J.E.-R.); john.montoya@uam.edu.co (J.M.-Z.); 4 Department of Animal Health, School of Veterinary Medicine, Faculty of Livestock Sciences, University of Nariño, Torobajo Campus, Pasto 520001, Colombia; astaizajm@gmail.com; 5 VITALAB Veterinary Diagnostics SAS, Ciudadela Complex, Lote 22 Llanogrande, Rionegro 054048, Colombia; direccionvitalab@gmail.com; 6 Department of Clinical Sciences, Faculty of Veterinary Medicine, University of Montreal, P.O. Box 6128, Saint-Hyacinthe, QC J2S 7C6, Canada; rejean.lefebvre@umontreal.ca

**Keywords:** cervix, cytology, cervicitis, endometritis, dairy cow, neutrophils

## Abstract

The bovine cervix undergoes endocrine-dependent physiological changes that may influence local immune activity and complicate the interpretation of cervical inflammation. This study evaluated the relationships among macroscopic cervical appearance, cervical neutrophil infiltration, reproductive stage, and endometrial inflammation in postpartum grazing dairy cows. Non-pregnant lactating Holstein × Jersey crossbred cows (*n* = 80) ≥ 45 days in milk underwent reproductive examination, cervical video-endoscopy, ultrasonography, cervical and endometrial cytology, and serum progesterone determination. Macroscopic cervical appearance was classified as C0, C1, or C2, whereas cervical polymorphonuclear neutrophil (PMN) percentage was analyzed as the primary proportional outcome using adjusted fractional logistic regression. A ≥5% cervical PMN threshold was used operationally for secondary analyses. C0, C1, and C2 occurred in 30.0%, 50.0%, and 20.0% of cows, respectively, and 20.0% had cervical PMN ≥ 5%. Macroscopic cervical appearance was not associated with cervical PMN percentage (*p* = 0.931) or cervical PMN ≥ 5% (*p* = 1.000). Cervical PMN percentage was higher in follicular-stage than in luteal-stage cows (*p* < 0.001). This association persisted after adjustment for parity and cytological endometritis using fractional logistic regression (β = 2.42; 95% CI, 0.81–4.03; *p* = 0.003), with an adjusted marginal difference of 10.48 percentage points (95% CI, 1.42–19.54; *p* = 0.023). A complementary two-part analysis supported this association, showing higher odds of detectable cervical PMNs (OR = 4.71; 95% CI, 1.73–12.82; *p* = 0.002) and higher mean PMN proportions among positive observations (mean ratio = 4.11; 95% CI, 1.40–12.06; *p* = 0.010) during the follicular stage. In secondary Firth penalized logistic regression, follicular stage remained associated with cervical PMN ≥ 5% (aOR = 5.38; 95% CI, 1.39–20.83; *p* = 0.015). Cervical and endometrial PMN percentages were weakly positively correlated (ρ = 0.240; *p* = 0.033), while agreement between cervical PMN ≥ 5% and cytological endometritis was limited (κ = 0.258). Neither cervical neutrophil infiltration nor cytological endometritis was associated with subsequent pregnancy, whereas serum progesterone concentration showed a positive exploratory association with pregnancy. Cervical neutrophil infiltration is associated with reproductive stage independently of parity and cytological endometritis, without relying on an unvalidated diagnostic threshold. Cervical cytology should therefore be interpreted within the reproductive and physiological context of the cow.

## Simple Summary

Inflammation of the cervix may be relevant to reproductive health in dairy cows, but interpreting cervical findings during routine reproductive examinations remains challenging. This study evaluated the relationships among macroscopic cervical appearance, cervical neutrophil infiltration, reproductive stage, and endometrial inflammation in postpartum grazing dairy cows. Macroscopic cervical appearance was not associated with cervical neutrophil infiltration, indicating that visible changes do not reliably reflect cytological findings. In contrast, cervical neutrophil infiltration was higher during the follicular stage than during the luteal stage. This association remained after accounting for parity and cytological endometritis and was supported by complementary statistical analyses that evaluated neutrophil infiltration without relying on a single diagnostic threshold. Cervical and endometrial neutrophil infiltration were weakly positively associated, although agreement between their cytological classifications was limited. No statistically significant association was detected between cervical neutrophil infiltration or cytological endometritis and subsequent pregnancy in this population. These findings highlight the importance of reproductive stage when interpreting cervical cytology and support evaluating neutrophil infiltration as a continuous measure rather than relying exclusively on an unvalidated diagnostic threshold for cervicitis.

## 1. Introduction

In dairy cattle, increasing milk yield is accompanied by elevated nutritional and metabolic demands, which predispose cows to postpartum uterine disorders [1]. These disorders negatively affect ovarian function, conception rates, and overall reproductive efficiency [2,3]. Despite substantial advances in the characterization of uterine disease, the diagnosis of postpartum inflammatory conditions of the reproductive tract remains challenging because inflammatory and physiological changes frequently coexist during routine postpartum examinations [4,5]. Discrepancies between clinical and cytological findings may partly reflect the complexity of inflammatory processes occurring at different anatomical compartments of the reproductive tract. In this context, the cervix has received comparatively limited attention in postpartum reproductive examinations. The cervix serves as a critical anatomical and functional barrier between the vagina and the uterus, preventing ascending infection while allowing regulated passage of spermatozoa and uterine secretions [6]. In addition to its mechanical barrier function, the cervix may participate actively in local immune regulation through interactions between epithelial cells, leukocytes, and cytokine signaling pathways. Cervicitis has been reported in postpartum dairy cows with variable prevalence depending on the diagnostic method and the postpartum interval evaluated. Hartmann et al. [6] reported clinical cervicitis in 60.8% of cows between 42 and 50 days postpartum, whereas Deguillaume et al. [7] reported increased neutrophil proportions in cervical cytology samples in up to 45% of postpartum cows. Additional evidence of cervical inflammation activity has been reported in cows presenting purulent discharge, elevated interleukin expression, and macroscopic cervical alterations [8,9,10]. However, these observations have generally been investigated independently, and it remains unclear whether macroscopic cervical appearance and cervical neutrophil infiltration represent the same biological process or distinct physiological and inflammatory components of postpartum cervical adaptation.

The cervix undergoes dynamic structural and physiological changes throughout the estrous cycle that are primarily regulated by fluctuations in ovarian steroid hormones. Estrogen predominance during the follicular phase promotes cervical relaxation, increased vascular perfusion, and abundant mucus secretion, whereas progesterone dominance during the luteal phase results in cervical tightening, reduced mucus hydration, and enhanced barrier function [11,12]. These endocrine-mediated changes regulate tissue vascularization, epithelial turnover, mucus characteristics, and immune activity within the reproductive tract [11]. Ovarian steroid hormones modulate leukocyte trafficking, cytokine production, and innate immune responses throughout the reproductive tract [2]. Therefore, endocrine-dependent physiological changes may influence both macroscopic cervical appearance and local neutrophil infiltration without necessarily indicating pathological inflammation. Despite increasing recognition of cervical inflammatory findings in postpartum dairy cows [6,7], the influence of reproductive stage on the interpretation of these findings has not been systematically investigated. Physiological changes in cervical vascularization, tone, and mucus production across reproductive stages may resemble inflammatory macroscopic changes, whereas endocrine regulation of immune cell migration may independently influence cervical neutrophil proportions. Consequently, macroscopic cervical appearance and cervical cytology may reflect related, but not necessarily equivalent, biological processes during postpartum reproductive examinations. In addition, cervical and endometrial inflammation may also coexist without necessarily representing equivalent inflammatory conditions. Deguillaume et al. [7], evaluating cervical and endometrial cytology simultaneously, identified cows with isolated endocervical inflammation, isolated endometrial inflammation, or both conditions, and reported that the presence or absence of cervical inflammation was not indicative of the corresponding endometrial status. Moreover, local inflammatory responses may involve multiple compartments of the reproductive tract. Vallejo-Timarán et al. [10] reported increased inflammatory mediator concentrations in the uterus and vaginal fornix of cows with clinical cervical alterations, particularly when accompanied by purulent vaginal discharge or subclinical endometritis. Together, these findings support biological interactions among inflammatory responses occurring at different sites of the postpartum reproductive tract.

Understanding how reproductive stage influences cervical morphology and cervical neutrophil infiltration is essential for the biological interpretation of postpartum reproductive examinations in dairy cows. This topic is particularly relevant under grazing systems managed in high-altitude tropical conditions, where information on postpartum cervical physiology and cervical inflammatory dynamics remains scarce. We hypothesized that reproductive stage would be associated with macroscopic cervical appearance and cervical neutrophil infiltration in postpartum dairy cows. Therefore, the primary objective of this study was to characterize macroscopic cervical appearance and cervical neutrophil infiltration in postpartum grazing dairy cows and to evaluate their relationship with reproductive stage. As an additional objective, we evaluated the relationship between cervical neutrophil infiltration and cytological endometritis and examined the agreement between cervical and endometrial cytological classifications.

## 2. Materials and Methods

### 2.1. Study Location and Herd Management

The study was conducted in the Nariño region, Pasto subregion, southwestern Colombia, within the dairy herd of the Obonuco Research Center of the Colombian Agricultural Research Corporation (AGROSAVIA), located in one of the main dairy-producing regions of the country. The production system is situated at 2905 m above sea level and is characterized by a mean annual precipitation of 619.7 mm, relative humidity of 84.4%, and mean ambient temperature of 12.8 °C. The feeding system is based on permanent rotational grazing, with rotation periods ranging from 25 to 45 days. Cows did not receive additional energy or protein supplementation, and mineral supplementation was provided through mineralized salt containing 6–8% phosphorus. Nutritional adjustments during the transition period were implemented as part of routine herd management. On average, approximately 70 cows were milked per month, with two daily milkings performed using a mechanized milking system. Animals included in the study were Holstein × Jersey crossbred cows and were maintained under the routine postpartum reproductive monitoring program implemented by the herd veterinarians.

### 2.2. Study Design

A cross-sectional observational study was conducted to evaluate the association of cervical neutrophil infiltration with macroscopic appearance, cytological endometritis, and reproductive stage during postpartum reproductive examinations. Each examination included clinical evaluation of the cervix by vaginoscopy and video-endoscopy, cervical and endometrial cytology, transrectal palpation and ultrasonography of the reproductive tract, and serum progesterone determination. These procedures allowed the simultaneous characterization of cervical morphology, cervical and endometrial inflammatory findings, and reproductive stage within the same reproductive examination. The relationship between cervical neutrophil infiltration and cytological endometritis, as well as the agreement between cervical and endometrial cytological classifications, was additionally evaluated.

### 2.3. Sample Size Calculation and Sampling Strategy

The original sample size was calculated to test the null hypothesis of equal proportions between cervical alterations identified by conventional examination (vaginoscopy) and cervical alterations identified in relation to estrous cycle stage. A two-sided test for two independent proportions was used, with a 95% confidence level (α = 0.05) and 80% statistical power (β = 0.20). Expected proportions were specified a priori as p_1_ = 0.80 for cervical alterations detected by conventional examination and p_2_ = 0.50 for cervical alterations identified in relation to estrous cycle stage. The assumption of a high frequency of cervical alterations detected by visual examination was informed by previous reports of frequent postpartum cervical alterations in dairy cows, including the 60.8% prevalence reported by Hartmann et al. [6]. The expected proportion of 0.50 for cervical alterations in relation to estrous cycle stage was adopted as a planning assumption because of the lack of previous evidence for this comparison. Sample size calculations were performed using Epitools–AusVet [13], following established recommendations for veterinary epidemiological study design [14,15]. A 10% allowance for potential animal or data losses was added, resulting in a target sample size of 80 cows. Because this original calculation was developed for a comparison of proportions, it did not constitute a formal power calculation for each of the subsequent analyses involving continuous cervical PMN percentages or individual reproductive stages.

Cows were therefore enrolled consecutively as calvings occurred over a seven-month study period until the target sample size was reached. Based on the herd’s historical calving and pregnancy records, approximately 15 postpartum cows were evaluated per month over a seven-month study period. A total of 115 postpartum dairy cows were initially screened, of which 80 cows ≥ 45 days in milk (DIM) met the eligibility criteria and were included in the study. Eligible animals were lactating, non-pregnant, Holstein × Jersey crossbred postpartum dairy cows. Exclusion criteria included dystocia, assisted calving or cesarean section, retained placenta, systemic disease (fever, anorexia, or recumbency), purulent vaginal discharge, and macroscopic cervical lesions detected during endoscopic examination. Anestrus was defined as the absence of a corpus luteum and the presence of ovarian follicles without diameter changes during two reproductive evaluations separated by an interval of eight days [16]. The 8-day interval between reproductive examinations was selected to allow confirmation of luteal activity and to reduce the probability of misclassification of anestrus or early luteal structures. No additional reproductive examinations were performed between the two scheduled examinations.

### 2.4. Clinical Examination and Sample Collection

Reproductive clinical examination included vaginoscopy, cervical video-endoscopy, transrectal palpation, transrectal ultrasonography, cervical and endometrial cytology, and blood sampling by coccygeal venipuncture for serum progesterone determination. Transrectal examination was used to evaluate uterine symmetry, uterine tone, uterine contents, and ovarian structures. Ultrasonographic examination (VINNO A5 scanner, Vinno Technology (Suzhou) Co., Ltd., Suzhou, China) was subsequently performed to confirm ovarian structures and characterize uterine findings. Vaginoscopy was performed using a vaginal speculum to visualize the cervix and identify cervical or uterine discharge. Following initial visualization, a 5.8-mm fiber-optic video-endoscope (TK-VETKIT–EC150H, Zhuhai TK VETKIT Medical Technology Co., Ltd., Zhuhai, China) was introduced to record cervical morphology (Supplementary Video S1). Cervical and endometrial cytological samples were obtained using the Cytobrush technique [17,18] (Supplementary Video S2).

Cervical samples were collected from the first cervical ring. Samples were fixed in 90% methanol and send to VITALAB SAS Diagnostic Veterinary Lab, where they were stained using Wright stain (Albor^®^ Biochemical, Bogotá, Colombia) and cytologically evaluated. Differential counts of 300 cells per slide were performed at 100× magnification to determine the proportion of polymorphonuclear neutrophils (PMN). Laboratory personnel were blinded to the clinical findings, macroscopic cervical appearance, and reproductive stage; samples were identified only by coded registration numbers. All cervical and endometrial cytology slides were evaluated twice by an experienced professional with more than 20 years of experience in cytological assessment. The second evaluation was performed without access to the results of the initial reading to assess intraobserver repeatability. In addition, a second professional from a different diagnostic laboratory independently evaluated the cytological slides once to assess interobserver agreement. Thus, intraobserver repeatability was based on two readings by the same observer, whereas interobserver agreement was based on independent assessments performed by two different professionals.

Blood samples were collected by coccygeal venipuncture for serum progesterone determination. Serum progesterone concentrations were measured using a competitive enzyme immunoassay (Progesterone AccuBind^®^ ELISA Test System, product code 4825-300, lot EIA-48K2C4; Monobind Inc., Lake Forest, CA, USA) and expressed in ng/mL. The assay was calibrated using standards containing 0, 0.3, 2, 5, 15, 30, and 60 ng/mL progesterone, with quality-control samples included in the analytical run. The analytical sensitivity reported by the manufacturer was 0.105 ng/mL. The kit identification and quality-control documentation correspond to the AccuBind^®^ ELISA system used in the study. The calibration report for the bovine progesterone assay confirms the measurement units and the seven calibration standards used for the analytical run.

### 2.5. Cervical Findings and Reproductive Stage Definitions

Macroscopic cervical appearance was assessed by vaginoscopy examination and classified into three cervical categories according to the degree of cervical congestion and protrusion of the second cervical ring [6]. C0 was defined as a pale-pink cervical mucosa without protrusion of the second cervical ring. C1 corresponded to a congested cervical mucosa without protrusion of the second cervical ring, whereas C2 was characterized by a congested cervical mucosa with protrusion of the second cervical ring. Macroscopic cervical appearance was analyzed as an ordinal variable (C0–C2).

Cervical neutrophil infiltration was quantified as the percentage of PMN among 300 cells evaluated in cervical cytology samples. Cervical PMN percentage was primarily analyzed as a continuous variable to avoid reliance on an unvalidated diagnostic cutoff. For secondary analyses, ≥5% PMN was used as an operational threshold based on previous studies of endocervical inflammation in postpartum dairy cows [7]. Because a validated diagnostic threshold for cervicitis has not been established for cows examined after 45 DIM, this cutoff was not interpreted as a definitive diagnosis of cervicitis. Sensitivity analyses were conducted using thresholds of ≥3% and ≥10% PMN to evaluate the robustness of findings to alternative definitions of increased cervical neutrophil infiltration. Endometrial neutrophil infiltration was quantified as the percentage of PMN in endometrial cytology samples. Cytological endometritis was defined as ≥5% PMN, based on previously established cytological criteria for postpartum dairy cows [17,19,20]. The term cytological endometritis was used to describe the cytological finding of increased endometrial neutrophil infiltration rather than the broader term subclinical endometritis, because classification in the present study was based specifically on endometrial cytology. Cows with <5% endometrial PMN were classified as negative for cytological endometritis.

Estrous cycle stage was determined based on clinical reproductive examination and ovarian ultrasonography. Metestrus was defined by an edematous and flaccid uterus together with a hemorrhagic body or a developing corpus luteum ≤ 6 mm with residual hemorrhagic characteristics. Diestrus was characterized by a moderately flaccid uterus and a corpus luteum > 16 mm. Proestrus was defined by increased uterine tone, the presence of a preovulatory follicle approximately 15–18 mm in diameter, occasionally accompanied by a regressing corpus luteum < 10 mm, and clear, translucent, filamentous cervicovaginal mucus. Estrus was characterized by increased uterine tone, a follicle > 12 mm, and absence of a corpus luteum. For analyses requiring broader reproductive-stage classification, metestrus and diestrus were grouped as the luteal stage, whereas proestrus and estrus were grouped as the follicular stage. Serum progesterone concentrations were used as complementary physiological information to refine the interpretation of reproductive stage in conjunction with the clinical and ultrasonographic findings, rather than as an independent classification criterion.

### 2.6. Reproductive Management

After the reproductive examination and sample collection, cows entered the reproductive management program of the herd. Cows classified as being in metestrus were monitored for subsequent natural estrus and inseminated following estrus detection. Cows classified as being in diestrus entered a fixed-time artificial insemination (FTAI) protocol immediately after the reproductive examination. On Day 0, an intravaginal progesterone-releasing device (Sincrogest^®^ 1 g; Ourofino, Brazil) was inserted and 2 mL of estradiol benzoate (Sincrodiol^®^ 1 mg; Ourofino, Brazil) was administered intramuscularly. Seven days later (Day 7), the intravaginal device was removed and 2 mL of sodium cloprostenol (Sincrocio^®^ 25 mg; Ourofino, Brazil) was administered intramuscularly. On Day 8, 1 mL of estradiol benzoate (Sincrodiol^®^ 1 mg; Ourofino, Brazil) was administered intramuscularly. Fixed-time artificial insemination was performed 52–54 h after removal of the intravaginal progesterone device. Cows classified as being in proestrus or estrus were inseminated following detection of natural estrus. Following artificial insemination, cows were monitored for return to estrus within the expected interval of 21 ± 2 days. Pregnancy diagnosis was performed 28 days after each insemination. Cows diagnosed as non-pregnant were re-enrolled in the reproductive management program through estrus detection and subsequent insemination or hormonal synchronization followed by insemination, as appropriate. This process continued for up to five insemination services per cow. Cows that failed to establish pregnancy by the fifth service were classified as non-pregnant for the reproductive outcome analysis. Following a positive pregnancy diagnosis, pregnancy maintenance was monitored at approximately 28-day intervals, with confirmation at approximately 120 days of gestation. Thus, the reproductive outcome represented pregnancy achieved and maintained within a management program allowing up to five services, rather than conception following a single insemination.

### 2.7. Statistical Analysis

Statistical analyses were performed using Stata/BE 19.5 (StataCorp LLC., College Station, TX, USA). Categorical variables were summarized as frequencies and percentages, whereas continuous variables were summarized using medians and interquartile ranges (IQR) when their distributions were asymmetric. For descriptive analyses, cows were classified into three reproductive categories: luteal stage (metestrus/diestrus; *n* = 42), follicular stage (proestrus/estrus; *n* = 34), and anestrus (*n* = 4). Differences in cervical PMN percentage among the three categories were initially evaluated using the Kruskal–Wallis test. The main inferential comparison of reproductive stage was restricted to cows classified in the luteal or follicular stages (*n* = 76); cows classified as anestrus were retained in the overall descriptive analyses but were not included in this comparison. Cervical PMN percentages between luteal- and follicular-stage cows were compared using the Wilcoxon rank-sum test. Serum progesterone concentrations were descriptively compared between the clinically defined follicular and luteal stages. Progesterone concentrations were summarized using medians and interquartile ranges, and differences between reproductive stages were assessed using the Mann–Whitney U test.

Cervical PMN percentage was considered the primary inflammatory outcome and was analyzed as a continuous variable. To evaluate the adjusted association between reproductive stage and the primary inflammatory outcome, fractional logistic regression was performed using cervical PMN proportion (percentage divided by 100) as the dependent variable. This approach was selected to accommodate the markedly right-skewed distribution and high frequency of zero values without dichotomizing the outcome. The model included reproductive stage as the main explanatory variable, with parity and cytological endometritis as covariates. Robust standard errors clustered by animal identification were used. Adjusted marginal mean PMN percentages and average marginal differences between reproductive stages were estimated with 95% confidence intervals (CI). As a complementary sensitivity analysis, a two-part model was fitted to account for the zero-heavy distribution of cervical PMN percentage. The first component used logistic regression to evaluate the presence of detectable cervical PMNs (>0%), whereas the second component used a gamma generalized linear model with a log link to evaluate PMN proportion among observations with positive values. Both components included reproductive stage, parity, and cytological endometritis, with robust standard errors clustered by animal identification.

For secondary analyses, cervical PMN percentage was dichotomized using an operational threshold of ≥5% PMN. The proportion of cows exceeding this threshold was compared between luteal and follicular stages using Fisher’s exact test, and crude odds ratios (OR) with 95% CI were estimated. Sensitivity analyses were conducted using alternative thresholds of ≥3% and ≥10% cervical PMN to determine whether the observed association was dependent on the selected operational cutoff. Associations between macroscopic cervical appearance (C0, C1, and C2) and continuous cervical or endometrial PMN percentages were evaluated using Kruskal–Wallis tests. Associations between macroscopic cervical appearance and dichotomous cytological classifications were evaluated using Fisher’s exact tests. The relationship between continuous cervical and endometrial PMN percentages was assessed using Spearman’s rank correlation coefficient. Cervical PMN percentages were also compared according to cytological endometritis status using the Wilcoxon rank-sum test. The association between cervical PMN ≥ 5% and cytological endometritis was evaluated using Fisher’s exact test, and agreement between the corresponding cervical and endometrial cytological classifications was quantified using Cohen’s kappa coefficient. Intraobserver repeatability was assessed by comparing two independent PMN percentage measurements performed by the same observer. Interobserver agreement was assessed by comparing the cytological measurements obtained by the first observer with those obtained independently by a second observer from another laboratory. Intraclass correlation coefficients (ICC) were used to quantify agreement in continuous cervical PMN percentages. For categorical agreement, each cervical PMN measurement was classified using the operational threshold of ≥5% PMN, and Cohen’s kappa coefficients were calculated separately for intraobserver and interobserver comparisons.

As a secondary multivariable analysis, Firth’s penalized logistic regression was used to estimate the association between reproductive stage and the operational outcome of cervical PMN ≥ 5%. The parsimonious model included reproductive stage as the exposure of interest, parity as an ordinal covariate, and cytological endometritis as a biologically relevant covariate. To assess potential confounding by postpartum timing, additional sensitivity analyses were performed using Firth’s penalized logistic regression with DIM as a continuous covariate, first in a model including reproductive stage and subsequently in a model additionally adjusted for parity and cytological endometritis. Adjusted OR and 95% CI were reported. Firth penalized logistic regression was applied consistently to the ≥3%, ≥5%, and ≥10% PMN classifications. All models included reproductive stage as the exposure of interest, with parity and cytological endometritis as covariates. Adjusted odds ratios and 95% confidence intervals were estimated to assess the consistency of the association across thresholds while reducing small-sample bias.

Subsequent pregnancy was analyzed in relation to serum progesterone concentration, cervical neutrophil infiltration (PMN ≥ 5%), cytological endometritis (endometrial PMN ≥ 5%), and endometrial PMN percentage as a continuous variable. Pregnancy proportions were compared using Fisher’s exact test, as appropriate. Associations between pregnancy and cervical PMN ≥ 5%, cytological endometritis, and continuous endometrial PMN percentage were evaluated using unadjusted logistic regression. The association between baseline serum progesterone concentration and subsequent pregnancy was evaluated using an exploratory logistic regression model adjusted for parity. Services per conception were summarized only among cows that became pregnant. Statistical significance was set at *p* < 0.05. Reproductive outcome analyses were considered exploratory because pregnancy status was determined within a stage-dependent reproductive management program allowing up to five insemination services per cow. The binary outcome was defined as pregnancy confirmed at approximately 120 days of gestation versus failure to achieve pregnancy by the fifth service. The progesterone model was not adjusted for reproductive stage or insemination protocol. Given the limited number of non-pregnant cows, no additional multivariable reproductive outcome models were fitted. Consequently, the reproductive associations were interpreted cautiously and were not considered evidence of independent causal effects.

### 2.8. Ethical Approval

All animal procedures were conducted in accordance with the experimental practices and standards approved by the AGROSAVIA Research Ethics Committee (Act No. 937, 31 May 2023). Cows were managed according to standard procedures for clinical examination and blood and reproductive sample collection, with measures taken to minimize adverse effects on animal welfare.

## 3. Results

### 3.1. Study Population

A total of 115 non-pregnant lactating dairy cows ≥ 45 DIM were screened for eligibility. Of these, 35 cows (30.4%) were excluded based on the predefined clinical eligibility criteria, and 80 cows (69.6%) were included in the study. Reasons for exclusion included dystocia, retained fetal membranes, clinical signs of systemic disease, purulent vaginal discharge (PVD), and macroscopic cervical lesions that precluded standardized classification within the C0–C2 categories. Anestrus was not considered an exclusion criterion, and cows classified as anestrus were retained in the study population. The study population comprised 29 (36.3%) primiparous cows, 22 (27.5%) cows with two to four calvings, 19 (23.8%) cows with five to six calvings, and 10 (12.5%) cows with seven or more calvings. Mean milk yield at the time of examination was 16 L/cow/day. The distribution of DIM according to reproductive stage is presented in Supplementary Table S1. Median serum progesterone concentrations were 5.75 ng/mL in follicular-stage evaluations (*n* = 33) and 7.19 ng/mL in luteal-stage evaluations (*n* = 39). Although the difference was not statistically significant (Mann–Whitney U test, *p* = 0.151), progesterone concentrations were numerically higher in the luteal group, consistent with the expected physiological pattern of greater progesterone secretion during the luteal phase.

### 3.2. Macroscopic Cervical Appearance and Cervical Neutrophil Infiltration

**Figure 1:**
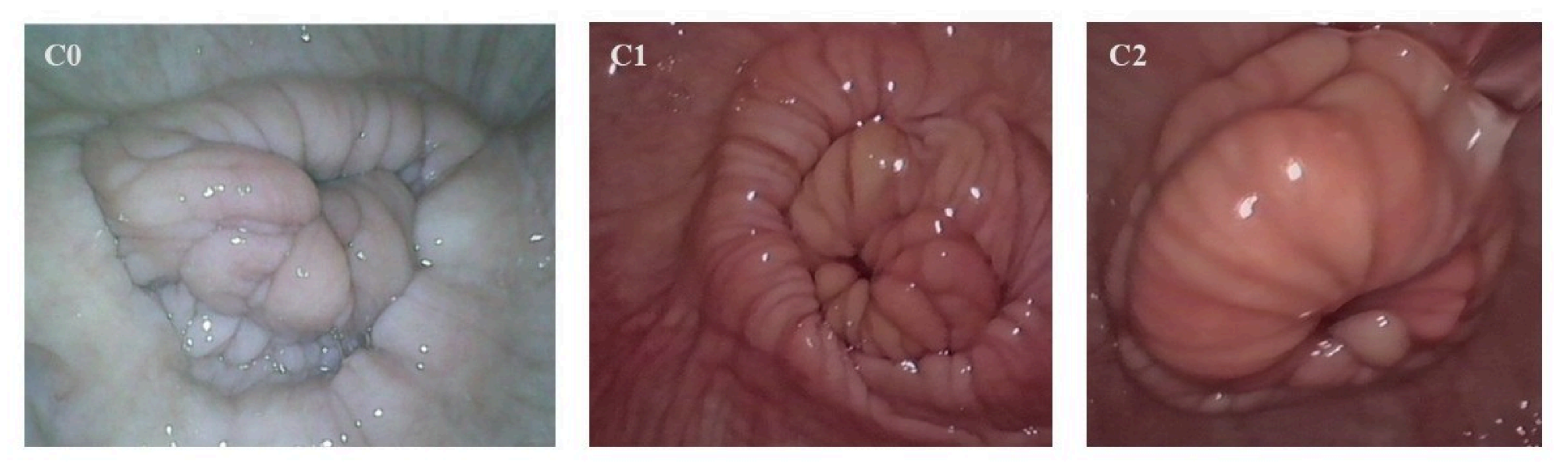
Clinical macroscopic characteristics of the cervix according to cervix grade in grazing dairy cows (*n* = 80) from high-tropics herds. Representative images of the three predefined cervical appearance categories used in the study: C0, pale pink cervical mucosa without protrusion of the second cervical ring; C1, congested cervical mucosa without protrusion of the second cervical ring; and C2, congested cervical mucosa with protrusion of the second cervical ring.

Clinical examination by vaginoscopy and video-endoscopy classified 24 of 80 cows (30.0%) as C0, 40 (50.0%) as C1, and 16 (20.0%) as C2 (Figure 1). Macroscopic cervical lesions that did not conform to the standardized C0–C2 classification (Cervix of different grades. Grade (G0)—Grade (G1)—Grade (G2) with the presence of macroscopic lesions in the mucosa and rings. Focal hemorrhagic areas in the mucosa (blue arrow); Cyanotic areas in the cervical rings (orange arrow); Thickening of the fornix (red arrow); Focal brown areas in the cervical rings.) are shown in Supplementary Figure S1.

Reproducibility of PMN assessment was evaluated for both the continuous PMN percentage and the corresponding positive/negative cytological classification. Intraobserver repeatability was evaluated using repeated cytological assessments performed by the same observer, yielding an ICC of 0.96 for continuous cervical PMN percentages and a Cohen’s kappa coefficient of 0.92 for the corresponding binary classifications. Interobserver agreement was assessed using an independent evaluation performed by a second professional from another laboratory, yielding an ICC of 0.92 and a Cohen’s kappa coefficient of 0.89.

**Figure 2:**
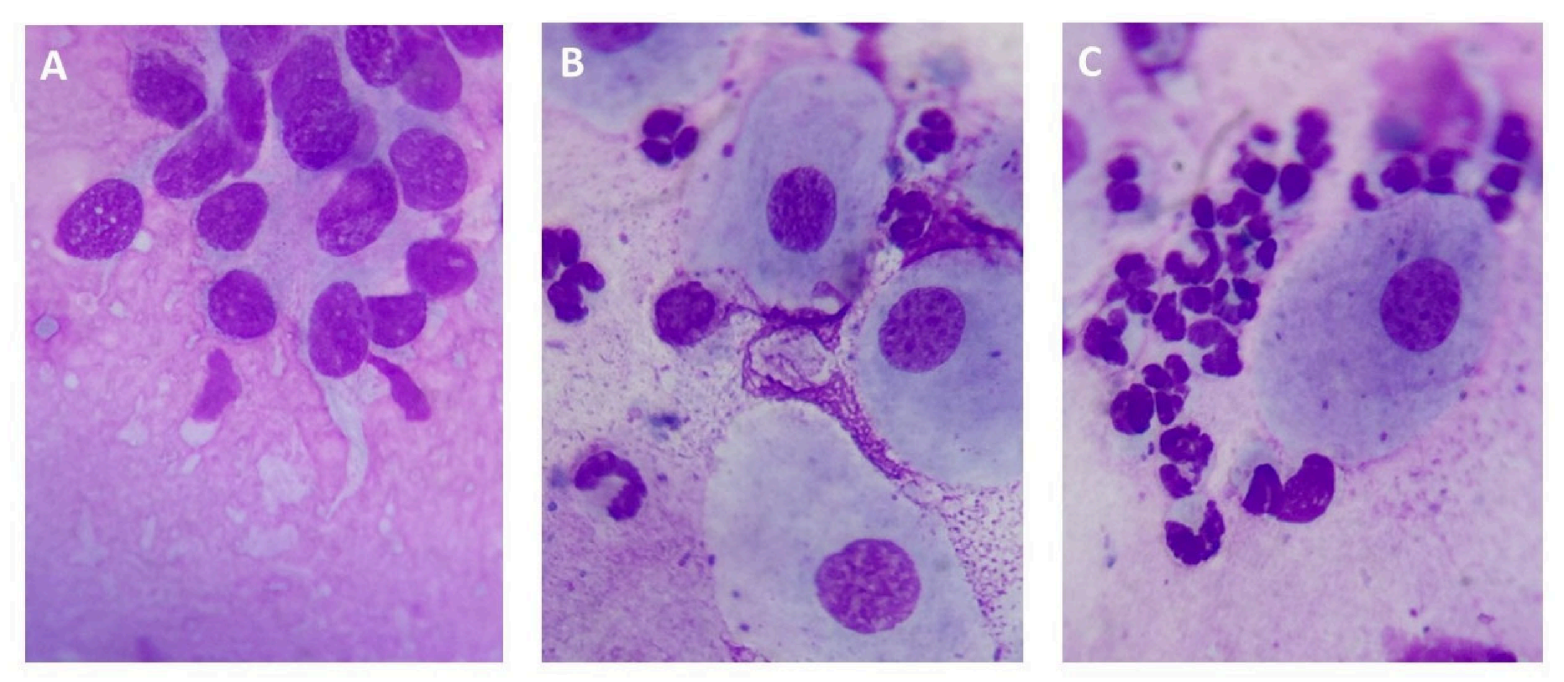
Cytological findings (Wright stain) of the cervix in grazing dairy cows (*n* = 80) from high-tropics herds. (**A**) Photomicrograph at 100× magnification of an accumulation of cervical wall epithelial cells that retain their cytomorphological integrity. Cells arranged mainly in sheet-like clusters (monolayers) of varying sizes and densities retain their cytomorphological integrity. (**B**,**C**) Photomicrograph at 100× magnification of cervical wall epithelial cells interspersed with abundant inflammatory response mediated by neutrophil polymorphonuclear leukocytes (some hypersegmented).

Cervical PMN percentage showed a markedly right-skewed distribution, with a median of 0% (IQR, 0–3%), a mean of 5.21% (SD, 14.10%), and a range of 0–74%. Using the ≥5% PMN operational threshold for secondary analyses, 16 of 80 cows (20.0%) had increased cervical neutrophil infiltration. Representative cervical cytology findings illustrating different degrees of neutrophil infiltration are shown in Figure 2. Macroscopic cervical appearance was not associated with cervical neutrophil infiltration. Cervical PMN percentages did not differ among C0, C1, and C2 categories (Kruskal–Wallis test, *p* = 0.931). Similarly, the proportion of cows with cervical PMN ≥ 5% was 20.8% (5/24), 20.0% (8/40), and 18.8% (3/16) in C0, C1, and C2, respectively, with no evidence of an association between macroscopic cervical category and the ≥5% PMN operational classification (Fisher’s exact test, *p* = 1.000).

### 3.3. Reproductive Stage and Cervical Neutrophil Infiltration

**Figure 3:**
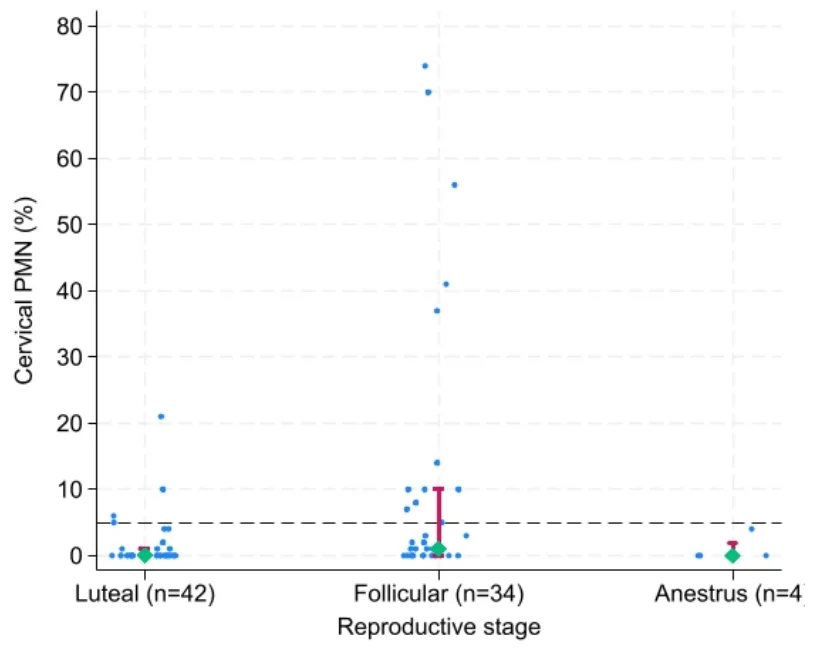
Distribution of cervical polymorphonuclear neutrophil (PMN) percentages according to reproductive stage in postpartum grazing dairy cows. Blue dots represent individual observations. The horizontal dashed line indicates the 5% PMN threshold.

From the 80 cows included in the study, 42 (52.5%) were classified in the luteal stage (metestrus/diestrus), 34 (42.5%) in the follicular stage (proestrus/estrus), and 4 (5.0%) as anestrus. Because only 16 of the 76 cows included in the luteal–follicular comparison had cervical PMN ≥ 5%, the adjusted binary analysis was considered secondary and exploratory. Cervical PMN percentages differed among the three reproductive categories (Kruskal–Wallis test, *p* = 0.003). Median cervical PMN percentage was 0% (IQR, 0–1%) in luteal-stage cows, 1% (IQR, 0–10%) in follicular-stage cows, and 0% (IQR, 0–2%) in anestrous cows (Table 1; Figure 3).

Individual observations are shown for cows classified in the luteal stage (*n* = 42), follicular stage (*n* = 34), or as anestrus (*n* = 4). Diamonds indicate medians and vertical bars represent interquartile ranges. Cervical PMN percentages differed among the three reproductive categories (Kruskal–Wallis test, *p* = 0.003). In the primary luteal–follicular comparison, cervical PMN percentage was higher in follicular-stage cows (Wilcoxon rank-sum test, *p* < 0.001). The horizontal dashed line indicates the ≥5% PMN operational threshold used in secondary analyses and does not represent a validated diagnostic cutoff for cervicitis.

The primary comparison between cows undergoing cyclic ovarian activity showed higher cervical PMN percentages in follicular-stage than in luteal-stage cows (Wilcoxon rank-sum test, *p* < 0.001). The four anestrous cows were retained in the overall study population and descriptive analysis but were not included in this luteal–follicular comparison. Using the ≥5% cervical PMN operational threshold, increased cervical neutrophil infiltration was observed in 4 of 42 luteal-stage cows (9.5%), 12 of 34 follicular-stage cows (35.3%), and none of the four anestrous cows. In the luteal–follicular comparison, cervical PMN ≥ 5% was more frequent during the follicular stage (Fisher’s exact test, *p* = 0.010), corresponding to a crude OR of 5.18 (95% CI, 1.49–18.04). Analyses using alternative operational thresholds of ≥3% and ≥10% cervical PMN showed the same direction of association (Table 1).

### 3.4. Relationship Between Cervical and Endometrial Neutrophil Infiltration

Cervical and endometrial PMN percentages showed a weak positive correlation (Spearman’s ρ = 0.240, *p* = 0.033). Cervical PMN percentages were also higher in cows with cytological endometritis than in cows without cytological endometritis (Wilcoxon rank-sum test, *p* = 0.011). Using the ≥5% cervical PMN operational threshold, 16 of 80 cows (20.0%) had increased cervical neutrophil infiltration, whereas cytological endometritis was identified in 25 of 80 cows (31.3%). Nine cows were positive according to both cytological classifications, seven had cervical PMN ≥ 5% without cytological endometritis, and 16 had cytological endometritis without cervical PMN ≥ 5% (Table 2). The two classifications were associated (Fisher’s exact test, *p* = 0.032); however, agreement between them was limited (Cohen’s κ = 0.258).

### 3.5. Macroscopic Cervical Appearance and Endometrial Neutrophil Infiltration

Macroscopic cervical appearance was not associated with endometrial neutrophil infiltration. Endometrial PMN percentages did not differ among cows classified as C0, C1, or C2 (Kruskal–Wallis test, *p* = 0.155). Cytological endometritis was identified in 4 of 24 C0 cows (16.7%), 14 of 40 C1 cows (35.0%), and 7 of 16 C2 cows (43.8%); however, the association between macroscopic cervical appearance and cytological endometritis was not statistically significant (Fisher’s exact test, *p* = 0.128).

### 3.6. Reproductive Stage and Cervical Neutrophil Infiltration

A total of 76 cows were included in the luteal–follicular comparison; of these animals, 16 had cervical PMN ≥ 5%. In the adjusted fractional logistic regression analysis of cervical PMN proportion (*n* = 76), follicular reproductive stage was positively associated with cervical neutrophil infiltration after adjustment for parity and cytological endometritis (β = 2.42; 95% CI: 0.81–4.03; *p* = 0.003). The average marginal difference in predicted cervical PMN percentage between follicular and luteal stages was 10.48 percentage points (95% CI: 1.42–19.54; *p* = 0.023). A complementary two-part analysis provided consistent findings. Follicular-stage evaluations had higher adjusted odds of detectable cervical PMNs (>0%) than luteal-stage evaluations (OR = 4.71; 95% CI: 1.73–12.82; *p* = 0.002). Among evaluations with detectable PMNs, the adjusted mean PMN proportion was also higher during the follicular stage (mean ratio = 4.11; 95% CI: 1.40–12.06; *p* = 0.010).

In the secondary Firth penalized logistic regression model adjusted for parity and cytological endometritis, follicular-stage cows had higher odds of cervical PMN ≥ 5% than luteal-stage cows (aOR = 5.38; 95% CI, 1.39–20.83; *p* = 0.015) (Table 3). Parity (aOR = 1.58; 95% CI, 0.87–2.89; *p* = 0.134) and cytological endometritis (aOR = 2.24; 95% CI, 0.69–7.21; *p* = 0.178) were not independently associated with cervical PMN ≥ 5%. The overall Firth model was statistically significant (Wald χ^2^ (3) = 9.26, *p* = 0.026). Sensitivity analyses using Firth penalized logistic regression showed associations in the same direction across the alternative operational cervical PMN thresholds. Compared with luteal-stage evaluations, follicular-stage evaluations had higher adjusted odds of cervical PMN ≥ 3% (aOR = 3.72; 95% CI: 1.14–12.12; *p* = 0.029) and ≥10% (aOR = 6.30; 95% CI: 1.28–31.02; *p* = 0.024). These findings were consistent with the primary Firth analysis using the ≥5% threshold (aOR = 5.38; 95% CI: 1.39–20.83; *p* = 0.015) (Table 3).

To assess potential confounding by postpartum timing, additional sensitivity analyses incorporating DIM were performed in the 66 luteal- and follicular-stage evaluations with complete DIM data (Supplementary Table S1). In Firth’s penalized logistic regression, the association between follicular stage and cervical PMN ≥ 5% persisted after adjustment for DIM (aOR = 3.94; 95% CI: 1.15–13.50; *p* = 0.029) and after additional adjustment for parity and cytological endometritis (aOR = 4.51; 95% CI: 1.09–18.64; *p* = 0.037). Similarly, fractional logistic regression adjusted for DIM, parity, and cytological endometritis confirmed the association between follicular stage and cervical PMN proportion (β = 2.405; 95% CI: 0.542–4.269; *p* = 0.011), with an adjusted marginal difference of 10.82 percentage points (95% CI: 0.04–21.59; *p* = 0.049). These findings support the consistency of the association after accounting for postpartum timing, although the reduced sample size should be considered when interpreting the estimates.

### 3.7. Reproductive Follow-Up

During reproductive follow-up, 65 of 80 cows (81.3%) achieved pregnancy confirmed at approximately 120 days of gestation within a reproductive management program allowing up to five insemination services. The remaining 15 cows (18.7%) were classified as non-pregnant after failing to achieve pregnancy by the fifth service. Among pregnant cows with available records (*n* = 64), the median number of services per conception was 1 (IQR, 1–2). In exploratory analyses, no statistically significant association was detected between subsequent pregnancy and cervical neutrophil infiltration (PMN ≥ 5%) or cytological endometritis (endometrial PMN ≥ 5%) (Table 4).

Pregnancy proportions were 75.0% and 82.8% in cows with and without cervical PMN ≥ 5%, respectively, and 80.0% and 81.8% in cows with and without cytological endometritis, respectively. No statistically significant association was detected between endometrial PMN percentage, analyzed as a continuous variable, and subsequent pregnancy (OR = 0.96; 95% CI: 0.91–1.01; *p* = 0.156). Baseline serum progesterone concentration showed a positive association with subsequent pregnancy in an exploratory model adjusted for parity (OR = 1.13 per 1 ng/mL increase; 95% CI: 1.01–1.26; *p* = 0.030). However, because reproductive management differed according to reproductive stage at the initial examination, this association cannot be interpreted independently of reproductive stage or the subsequent insemination protocol.

## 4. Discussion

The present study evaluated the relationships among reproductive stage, macroscopic cervical appearance, cervical neutrophil infiltration, and endometrial inflammation in postpartum grazing dairy cows. Three main findings emerged. First, cervical neutrophil infiltration was associated with reproductive stage, with higher cervical PMN percentages and a greater frequency of increased neutrophil infiltration in follicular-stage than in luteal-stage cows. This association remained after adjustment for parity and cytological endometritis and was consistent across alternative operational PMN thresholds. Second, macroscopic cervical appearance was not associated with cervical neutrophil infiltration, indicating that visible cervical changes and cytological neutrophil infiltration represent distinct findings in this population. Third, cervical and endometrial neutrophil infiltration were positively associated, but the weak correlation and limited agreement between their cytological classifications indicate that findings in these two anatomical compartments should not be considered interchangeable. Together, these results emphasize the importance of reproductive stage when interpreting cervical cytology and support evaluating cervical neutrophil infiltration independently from macroscopic cervical appearance and endometrial cytology.

### 4.1. Reproductive Stage and Cervical Neutrophil Infiltration

The main finding of the present study was the association between reproductive stage and cervical neutrophil infiltration. Cervical PMN percentages were higher in follicular-stage than in luteal-stage cows, and cervical PMN ≥ 5% was observed in 35.3% and 9.5% of cows, respectively. This difference was evident when cervical PMN was analyzed as a continuous variable, avoiding dependence on a single cytological threshold. In the multivariable analysis, follicular-stage cows had greater odds of cervical PMN ≥ 5% than luteal-stage cows after adjustment for parity and cytological endometritis. Moreover, sensitivity analyses using ≥3%, ≥5%, and ≥10% cervical PMN thresholds showed associations in the same direction. These findings suggest that reproductive stage is associated with variation in cervical neutrophil infiltration and should therefore be considered when interpreting cervical cytology. The association between reproductive stage and cervical neutrophil infiltration persisted when cervical PMN percentage was evaluated as the primary proportional outcome using adjusted fractional logistic regression. Follicular-stage evaluations showed a higher predicted cervical PMN percentage than luteal-stage evaluations, with an adjusted marginal difference of 10.48 percentage points after accounting for parity and cytological endometritis. This finding indicates that the association was not dependent on dichotomizing cervical PMN percentage using the operational ≥5% threshold. The complementary two-part analysis provided consistent evidence, showing that follicular-stage evaluations had both higher odds of detectable cervical PMNs and higher mean PMN proportions among positive observations. Together, these findings support an association between reproductive stage and cervical neutrophil infiltration across different analytical approaches, without establishing a causal relationship. Reproductive stage classification in the present study relied primarily on clinical and ultrasonographic findings, with serum progesterone providing complementary physiological information. Although progesterone concentrations did not differ significantly between the clinically defined follicular and luteal groups, the numerically higher median concentration observed during the luteal stage was consistent with the expected endocrine activity of the corpus luteum. Because reproductive-stage classification integrated multiple clinical and physiological criteria, progesterone concentrations were not intended to function as an independent diagnostic test for distinguishing reproductive stages.

The biological mechanisms underlying this association cannot be established from the present cross-sectional study. Nevertheless, endocrine–immune interactions within the bovine reproductive tract provide a plausible biological framework. Ovarian steroids regulate epithelial function, tissue remodeling, vascular characteristics, mucus properties, and local immune responses throughout the reproductive cycle. Estrogen predominance during the follicular stage is associated with cervical relaxation, increased vascular perfusion, and mucus secretion, whereas progesterone predominance during the luteal stage is associated with cervical closure and reduced mucus hydration [2,11]. Ovarian steroids may also modulate leukocyte trafficking, cytokine production, and innate immune responses within reproductive tissues [2,11,12]. Herath et al. [11] demonstrated innate immune capabilities in bovine ovarian follicular cells and interactions between innate immune signaling and endocrine function, providing further evidence of the close relationship between reproductive physiology and immune regulation. Thus, differences in cervical neutrophil distribution between follicular and luteal stages may reflect physiological reproductive–immune modulation of the cervical environment.

The greater cervical neutrophil infiltration observed during the follicular stage should not, however, be interpreted as evidence that follicular-stage hormones directly induce cervical inflammation. The present study identified an association between reproductive stage and cervical neutrophil distribution and cannot distinguish physiological leukocyte trafficking from pathological inflammation or establish a direct hormonal mechanism. This distinction is particularly relevant because the ≥5% cervical PMN threshold has not been validated as a diagnostic cutoff for cervicitis in cows examined after 4 5 DIM. Deguillaume et al. [7] evaluated endocervical inflammation according to postpartum interval and used ≥5% neutrophils before 35 DIM, a substantially earlier postpartum period than that evaluated in the present study. Although the association with reproductive stage remained consistent across alternative operational thresholds, this consistency supports the robustness of the observed association rather than the diagnostic validity of any cutoff. Longitudinal studies evaluating the same cows across successive stages of the estrous cycle are needed to determine whether cervical neutrophil trafficking follows a reproducible physiological pattern.

### 4.2. Macroscopic Cervical Findings and Cervical Neutrophil Infiltration

Macroscopic cervical appearance was not associated with cervical neutrophil infiltration in the present study. The proportion of cows with cervical PMN ≥ 5% was nearly identical among the C0, C1, and C2 categories (20.8%, 20.0%, and 18.8%, respectively), and cervical PMN percentages analyzed as a continuous variable likewise did not differ among macroscopic categories. These findings indicate that increasing macroscopic cervical alteration, as defined by congestion and protrusion of the second cervical ring, was not accompanied by greater neutrophil infiltration. Therefore, macroscopic cervical appearance and cervical cytology appear to represent distinct features of the postpartum cervix rather than interchangeable indicators of cervical inflammation. This distinction is relevant when comparing the present findings with previous studies because different approaches have been used to characterize cervical inflammation in postpartum dairy cows. Hartmann et al. [6] classified clinical cervicitis according to macroscopic cervical findings and reported a prevalence of 60.8% in cows examined approximately 6–7 weeks postpartum, whereas Deguillaume et al. [7] evaluated endocervical inflammation using neutrophil proportions. These approaches assess different biological features and were developed using different case definitions and postpartum intervals. Bazzazan et al. [21] further demonstrated that clinically diagnosed cervicitis may coexist with other postpartum reproductive tract disorders, emphasizing the complexity of interpreting macroscopic findings across different compartments of the reproductive tract. Consequently, prevalence estimates based on clinical appearance should not be directly equated with estimates based on cervical cytology.

The lack of correspondence between macroscopic appearance and cervical PMN infiltration is also biologically plausible. The cervix is a dynamic tissue whose vascularity, tone, mucus production, epithelial characteristics, and degree of opening vary with reproductive physiology. Thus, cervical congestion or protrusion may occur as part of physiological tissue remodeling without a corresponding increase in luminal neutrophils. Conversely, increased neutrophil infiltration may occur without marked changes detectable by visual examination. Our findings therefore support describing C0–C2 as categories of macroscopic cervical appearance rather than interpreting C2 itself as evidence of inflammatory cervicitis. Similarly, cervical PMN ≥ 5% should be described as an operational indicator of increased neutrophil infiltration rather than as a definitive diagnosis of cervicitis.

The frequency of cervical PMN ≥ 5% observed in the present study (20.0%) should also be interpreted within the characteristics of the study population. Cows were examined at ≥45 DIM, and animals with PVD, systemic disease, or macroscopic cervical lesions that precluded standardized C0–C2 classification were excluded. Therefore, this value represents the frequency of increased cervical neutrophil infiltration in the selected study population rather than an estimate of herd-level cervicitis prevalence. Video-endoscopy additionally allowed identification of focal hemorrhagic or cyanotic areas and structural abnormalities that could not be accommodated within the predefined C0–C2 categories. Distinguishing these lesions from the standardized macroscopic categories reduced the possibility of classifying visually abnormal but potentially traumatic, vascular, or structural findings solely as inflammatory changes. However, because the study was not designed to compare diagnostic methods, no conclusions can be drawn regarding the diagnostic superiority of video-endoscopy over conventional vaginoscopy.

### 4.3. Relationship Between Cervical and Endometrial Neutrophil Infiltration

Cervical and endometrial neutrophil infiltration were positively associated in the present study, although the magnitude of this relationship was limited. Cervical and endometrial PMN percentages showed a weak positive correlation (ρ = 0.240), and cervical PMN percentages were higher in cows with cytological endometritis. Similarly, cervical PMN ≥ 5% was associated with cytological endometritis. However, agreement between the two dichotomous classifications was limited (κ = 0.258). Of the 80 cows examined, only nine were positive in both compartments, whereas seven had cervical PMN ≥ 5% without cytological endometritis and 16 had cytological endometritis without cervical PMN ≥ 5%. Thus, although inflammatory findings in the cervix and endometrium were related, cervical and endometrial cytology did not identify the same animals consistently.

Previous studies support the concept that inflammatory responses may occur concurrently or independently in different compartments of the postpartum reproductive tract. Deguillaume et al. [7] reported that endocervical inflammation may occur either in association with or independently of endometrial inflammation, indicating that cervical cytology cannot be inferred directly from uterine findings. Likewise, evidence of local inflammatory activity in the bovine cervix, including changes in inflammatory mediators associated with clinical cervicitis, supports the biological capacity of the cervical compartment to develop a local immune response [10]. The anatomical continuity between the uterus and cervix provides a plausible basis for some degree of concordant inflammatory activity, but differences in epithelial characteristics, local immune regulation, microbial exposure, and resolution of postpartum inflammation may contribute to discordant cytological findings between these sites.

These results have implications for the interpretation of postpartum reproductive cytology. The significant association between cervical and endometrial neutrophil infiltration should not be interpreted as evidence that one measurement can substitute for the other. In particular, the weak correlation and limited agreement observed here indicate that endometrial cytology cannot reliably predict cervical neutrophil infiltration, and cervical cytology should not be considered a surrogate for cytological endometritis. Evaluation of the two anatomical compartments therefore provides complementary rather than interchangeable information. Further longitudinal studies combining cervical and endometrial cytology with microbiological and local inflammatory markers are needed to determine whether discordant findings represent differences in the onset, persistence, or resolution of inflammatory responses within the postpartum reproductive tract.

### 4.4. Cytological Findings and Reproductive Follow-Up

Reproductive follow-up provides additional context for interpreting the cytological findings, although these findings should be considered exploratory. In the present study, no statistically significant association was detected between cervical neutrophil infiltration or cytological endometritis and subsequent pregnancy. A similar result was observed when endometrial PMN percentage was analyzed as a continuous variable. However, these findings should not be interpreted as evidence that cervical or endometrial inflammation has no effect on fertility. Only 15 cows were classified as non-pregnant, limiting the statistical precision and the ability to detect potentially relevant reproductive associations. An important consideration is that reproductive management differed according to reproductive stage at the initial examination. Cows classified as being in diestrus entered a fixed-time artificial insemination protocol, whereas cows in other reproductive stages were inseminated following estrus detection. Subsequent pregnancy therefore reflected not only baseline reproductive and inflammatory characteristics but also stage-dependent reproductive management and repeated insemination opportunities, up to a maximum of five services. Furthermore, pregnancy was evaluated as a binary outcome rather than as time to conception, and the interval required to achieve pregnancy could differ among cows. The exploratory positive association between baseline serum progesterone concentration and subsequent pregnancy also requires caution. Progesterone concentration is closely related to reproductive stage, which influences the subsequent insemination protocol. Consequently, the observed association cannot be interpreted as an independent biological effect of progesterone on fertility. It may partly reflect differences in ovarian status, reproductive management, or other unmeasured factors. Previous studies have reported associations between reproductive tract inflammation and reduced reproductive performance. Madoz et al. identified impaired reproductive performance in grazing dairy cows classified with subclinical endometritis and proposed PMN thresholds varying according to postpartum interval [22], whereas Lee et al. reported a higher endometrial PMN threshold (≥14%) at four weeks postpartum associated with reduced subsequent reproductive performance [23]. Differences in study populations, postpartum intervals, cytological definitions, reproductive management, and outcome assessment may contribute to variation among findings. Therefore, the absence of a statistically significant association in the present population should be interpreted cautiously and does not exclude clinically meaningful effects of cervical or endometrial inflammation on fertility.

### 4.5. Strengths and Limitations

The present study combined the assessment of macroscopic cervical appearance, cervical and endometrial cytology, ovarian structures, and serum progesterone within the same reproductive examination. Cervical PMN percentage was evaluated as the primary proportional outcome using adjusted fractional logistic regression, allowing the association with reproductive stage to be assessed without relying on an unvalidated diagnostic cutoff. The complementary two-part model further addressed the substantial proportion of zero values by separately evaluating the presence and magnitude of cervical neutrophil infiltration. Secondary analyses using operational thresholds of ≥3%, ≥5%, and ≥10% PMN provided additional evidence regarding the consistency of the association across different definitions of increased neutrophil infiltration. In addition, cytological slides were evaluated under blind conditions, with high intra- and interobserver repeatability. Several limitations should also be considered. The cross-sectional design did not allow the same cows to be followed through successive reproductive stages. The observed differences therefore represent between-cow associations and cannot establish within-cow cyclical changes or causal relationships between reproductive stage and cervical neutrophil infiltration. Longitudinal studies sampling the same cows across consecutive stages of the estrous cycle would be particularly valuable. Only 16 cows in the primary luteal–follicular analysis had cervical PMN ≥ 5%, limiting the precision of multivariable analyses of this binary outcome. Although fractional logistic regression allowed cervical PMN percentage to be evaluated without dichotomization, the relatively small sample size and highly asymmetric distribution of PMN values may affect the precision of adjusted estimates. The adjusted Firth model was therefore deliberately parsimonious, and its relatively wide confidence interval indicates that the magnitude of the association should be interpreted cautiously. The ≥5% cervical PMN threshold should be considered an operational indicator of increased neutrophil infiltration rather than a validated diagnostic cutoff for cervicitis in cows evaluated after 45 DIM. Previous evidence supporting the use of this threshold was generated under different postpartum conditions, including earlier postpartum intervals [7]. Given the variability in postpartum timing. DIM was considered a potential confounding factor. Sensitivity analyses incorporating DIM as a continuous covariate showed that the association between reproductive stage and cervical PMN ≥ 5% persisted after adjustment for postpartum timing. Although sensitivity analyses using alternative thresholds yielded associations in the same direction, these findings do not validate any specific cutoff. Although cows were managed under a program allowing up to five insemination services, the timing and type of reproductive intervention depended on reproductive stage at baseline. The limited number of non-pregnant cows (n = 15), the use of a binary pregnancy outcome, and the absence of adjustment for reproductive stage or insemination protocol restrict the interpretation of the exploratory reproductive associations, particularly that involving serum progesterone. Studies integrating cervical cytology with histopathology, local inflammatory mediators, and microbiological or microbiome characterization are needed to establish biologically and clinically meaningful reference values for cervical PMN infiltration. Finally, the study was conducted in a single high-altitude grazing herd and included a selected population of clinically stable postpartum cows after predefined exclusions. Consequently, the findings may not be directly generalizable to herds differing in breed composition, production intensity, postpartum disease burden, environmental conditions, nutrition, or reproductive management. Residual confounding by cow-level factors not fully accounted for in the present analysis also cannot be excluded.

## 5. Conclusions

Cervical neutrophil infiltration in postpartum grazing dairy cows was associated with reproductive stage but not with macroscopic cervical appearance. Follicular-stage evaluations showed higher cervical PMN percentages than luteal-stage evaluations, and this association persisted in adjusted fractional logistic regression after accounting for parity and cytological endometritis. The complementary two-part analysis supported this finding, indicating higher odds of detectable cervical PMNs and greater neutrophil infiltration among positive observations during the follicular stage. Secondary analyses using operational PMN thresholds yielded associations in the same direction. Cervical and endometrial neutrophil infiltration were positively associated, but their weak correlation and limited agreement indicate that cytological findings from these anatomical compartments are related but not interchangeable. The association between reproductive stage and cervical PMN ≥ 5% persisted in sensitivity analyses accounting for DIM, parity, and cytological endometritis. These results support interpreting cervical PMN percentage in the context of reproductive stage and as a continuous measure rather than relying solely on an unvalidated diagnostic threshold. Macroscopic cervical appearance should likewise not be considered a surrogate for cervical neutrophil infiltration. No statistically significant association was detected between cervical or endometrial neutrophil infiltration and subsequent pregnancy in this population. However, these exploratory findings should be interpreted cautiously because of the limited number of non-pregnant cows and differences in reproductive management according to baseline reproductive stage. Further studies with standardized reproductive protocols and longitudinal fertility outcomes are needed to clarify the reproductive significance of cervical neutrophil infiltration. Longitudinal studies are needed to characterize physiological variation in cervical neutrophil trafficking across the reproductive cycle and to establish clinically meaningful reference values for cervical cytology in postpartum dairy cows.

## Figures and Tables

**Table 1:** Cervical neutrophil infiltration according to reproductive stage in postpartum grazing dairy cows.

Cervical PMN	Luteal Stage (*n* = 42)	Follicular Stage (*n* = 34)	Anestrus (*n* = 4)	*p*
PMN, %, median (IQR)	0 (0–1)	1 (0–10)	0 (0–2)	0.003 ᵃ
PMN ≥ 3%, *n* (%)	6 (14.3)	14 (41.2)	1 (25.0)	0.010 ᵇ
PMN ≥ 5%, *n* (%)	4 (9.5)	12 (35.3)	0 (0.0)	0.010 ᵇ
PMN ≥ 10%, *n* (%)	2 (4.8)	9 (26.5)	0 (0.0)	0.010 ᵇ

PMN= polymorphonuclear neutrophils; IQR= interquartile range. ᵃ *p* value from the Kruskal–Wallis test comparing cervical PMN percentages across the three reproductive categories (luteal stage, follicular stage, and anestrus). ᵇ *p* values from Fisher’s exact tests comparing luteal- and follicular-stage cows only; anestrous cows were retained for descriptive purposes but were not included in these comparisons. The ≥3%, ≥5%, and ≥10% cervical PMN thresholds were used as operational thresholds for secondary and sensitivity analyses and should not be interpreted as validated diagnostic cutoffs for cervicitis.

**Table 2:** Relationship between cervical neutrophil infiltration and cytological endometritis in postpartum grazing dairy cows.

Cervical PMN%	Non-CYTO	CYTO	Total
<5% PMN	48 (75.0%)	16 (25.0%)	64
≥5% PMN	7 (43.8%)	9 (56.3%)	16
Total	55	25	80

PMN, polymorphonuclear neutrophils. Cytological endometritis was defined as endometrial PMN ≥ 5%. Cervical PMN ≥ 5% represents the operational threshold used for secondary analyses and should not be interpreted as a validated diagnostic cutoff for cervicitis. Percentages are calculated within cervical PMN classification rows. Fisher’s exact test, *p* = 0.032; Cohen’s κ = 0.258.

**Table 3:** Adjusted associations between reproductive stage and cervical neutrophil infiltration in grazing dairy cows from high-tropical herds.

Analysis/Predictor	Estimate	95% CI	*p*-Value
Primary analysis: Fractional logistic regression			
Follicular vs. luteal stage (β)	2.42	0.81–4.03	0.003
Adjusted marginal difference (percentage points)	10.48	1.42–19.54	0.023
Sensitivity analysis: Two-part model			
Presence of cervical PMNs (>0%), follicular vs. luteal stage (OR)	4.71	1.73–12.82	0.002
Positive cervical PMN proportion, follicular vs. luteal stage (MR)	4.11	1.40–12.06	0.010
Secondary analysis: Firth penalized logistic regression, PMN ≥ 5%			
Follicular vs. luteal stage (aOR)	5.38	1.39–20.83	0.015
Parity (aOR)	1.58	0.87–2.89	0.134
Cytological endometritis (aOR)	2.24	0.69–7.21	0.178
Sensitivity analyses: Alternative operational PMN thresholds (Firth penalized logistic regression)			
PMN ≥ 3%, follicular vs. luteal stage (aOR)	3.72	1.14–12.12	0.029
PMN ≥ 5%, follicular vs. luteal stage (aOR)	5.38	1.39–20.83	0.015
PMN ≥ 10%, follicular vs. luteal stage (aOR)	6.30	1.28–31.02	0.024

PMN, polymorphonuclear neutrophils; CI, confidence interval; OR, odds ratio; aOR, adjusted odds ratio; β, regression coefficient; MR, mean ratio. Analyses were restricted to cows classified in the follicular or luteal reproductive stages (*n* = 76); four anestrous cows were excluded. The primary fractional logistic regression model included reproductive stage, parity, and cytological endometritis, with robust standard errors clustered by animal identification. The adjusted marginal difference represents the average difference in predicted cervical PMN percentages between reproductive stages, expressed in percentage points. The complementary two-part model comprised logistic regression for detectable cervical PMNs (>0%; *n* = 76) and a gamma generalized linear model with a log link for positive cervical PMN proportions (*n* = 32). Both components were adjusted for reproductive stage, parity, and cytological endometritis, with robust standard errors clustered by animal identification. The secondary analysis of cervical PMN ≥ 5% and the sensitivity analyses using alternative thresholds (≥3% and ≥10%) were performed using Firth’s penalized logistic regression, adjusting for reproductive stage, parity (ordinal), and cytological endometritis. The overall model for cervical PMN ≥ 5% yielded Wald χ^2^ (3) = 9.26 (*p* = 0.026). All cervical PMN thresholds were operational and should not be interpreted as validated diagnostic cutoffs for cervicitis in postpartum dairy cows.

**Table 4:** Exploratory associations between baseline cervical and endometrial inflammatory findings, serum progesterone concentration, and pregnancy confirmed within a reproductive management program allowing up to five insemination services in postpartum grazing dairy cows.

Variable	Pregnancy *n*/*N* (%)	OR (95% CI)	*p*-Value
Cervical Cytology			
PMN < 5%	53/64 (82.8%)	Reference	-
PMN ≥ 5%	12/16 (75.0%)	0.62 (0.17–2.30)	0.477
Cytological endometritis			
No	45/55 (81.8%)	Reference	-
Yes	20/25 (80.0%)	0.89 (0.27–2.94)	0.847
Endometrial PMN count	-	0.96 (0.91–1.01)	0.156
Serum P4 (ng/mL) *	-	1.13 (1.01–1.26)	0.030

OR = odds ratio; CI = confidence interval; PMN = polymorphonuclear neutrophils; P4 = progesterone n, number of pregnant cows; N, total number of cows in each category. Cytological endometritis was operationally defined as endometrial PMN ≥ 5%. Pregnancy was assessed after each insemination and confirmed at approximately 120 days of gestation. Cows failing to achieve pregnancy by the fifth service were classified as non-pregnant. Reproductive management differed according to reproductive stage at baseline. Progesterone OR was obtained from an exploratory logistic regression model adjusted for parity but not for reproductive stage or insemination protocol. All other ORs shown are unadjusted. These analyses were exploratory and should not be interpreted as causal estimates. * Progesterone OR was obtained from the exploratory logistic regression model adjusted for parity. All other ORs shown are unadjusted.

## Data Availability

The data sets generated during the current study are available from the corresponding author upon reasonable request.

## Supplementary Materials

The following supporting information can be downloaded at: https://www.mdpi.com/article/10.3390/ani16213343/s1, Video S1: Cervical video-endoscopic examination in a postpartum dairy cow; Video S2: Collection of cervical and endometrial cytology samples using the cytobrush technique; Figure S1: Macroscopic findings in cervix (Different from clinical macroscopic characteristics of the cervix according to cervix grade) in grazing dairy cows (*n* = 80) from high-tropics herds. Table S1: Days in milk according to reproductive stage and DIM-adjusted sensitivity analyses of cervical neutrophil infiltration in postpartum grazing dairy cows.

## Abbreviations

The following abbreviations are used in this manuscript:

DIM	Days in milk
PMN	Polymorphonuclear neutrophils
PVD	Purulent vaginal discharge
P4	Progesterone
ELISA	Enzyme-linked immunosorbent assay
ICC	Intraclass correlation coefficient
IQR	Interquartile range
CI	Confidence interval
