# Supplementary material for: Cervical Neutrophil Infiltration in Relation to Macroscopic Cervix Appearance, Cytological Endometritis, and Reproductive Stage in Postpartum Grazing Dairy Cows
Source: Animals (Basel). 2026 Oct 23;16(21):3343. doi: 10.3390/ani16213343 (PMC13762291; doi:10.3390/ani16213343)
Supplement: Supplementary file 1 [file animals-16-03343-s001.zip › Supplementary_Table S1_DIM_FINAL.pdf]

Supplementary Table S1. Days in milk according to reproductive stage and DIM-adjusted sensitivity analyses of cervical neutrophil infiltration in postpartum grazing dairy cows.

	**Reproductive stage**	**DIM, median (IQR), days**	**DIM, range, days**
Distribution of days in milk (DIM) by reproductive stage	Luteal (metestrus/diestrus)	124 (92–198)	51–415
	Follicular (proestrus/estrus)	133 (99–187)	75–411
	Anestrus	125.5 (85–184.75)	82–244
	Overall	125.5 (95.5–197)	51–415
	**Model / predictor**	**Adjusted OR (95% CI)**	**P value**
DIM-adjusted sensitivity analysis: Firth penalized logistic regression (outcome: cervical PMN ≥5%)	Model 1: reproductive stage + DIM		
	Follicular vs. luteal stage	3.939 (1.150–13.499)	0.029
	DIM (per 10 additional days)	1.000 (0.941–1.063)	0.9997
	Model 2: stage + DIM + parity + cytological endometritis		
	Follicular vs. luteal stage	4.515 (1.093–18.641)	0.037
	DIM (per 10 additional days)	0.997 (0.932–1.068)	0.938
	Parity (one-category increase)	1.608 (0.799–3.233)	0.183
	Cytological endometritis (yes vs. no)	2.449 (0.672–8.928)	0.175
	**Analysis / predictor**	**Estimate** 95% CI	**P value**
DIM-adjusted primary-outcome sensitivity analysis: fractional logistic regression (cervical PMN proportion)	Follicular vs. luteal stage (β)	2.405 (0.542–4.269)	0.011
	Average adjusted marginal difference (percentage points)	10.82 (0.04–21.59)	0.049

DIM, days in milk; PMN, polymorphonuclear neutrophils; IQR, interquartile range; OR, odds ratio; CI, confidence interval. The analysis merged cervical cytology records with examination dates and the most recent preceding calving dates identified in the herd diary, matched by evaluation number, cow ID, and date. DIM was available for 70 of 80 assessments; the 10 assessments without a documented preceding calving date were not imputed. Binary-model analyses are restricted to the 66 complete-case luteal/follicular assessments (14 PMN ≥5% observations; 44 distinct cow IDs). The reference categories are luteal stage and absence of cytological endometritis. DIM was modeled continuously and ORs are presented per 10-day increase. Firth penalized logistic regression estimates use model-based Wald confidence intervals and P values; the primary-outcome fractional logistic sensitivity model additionally adjusted for parity and cytological endometritis and used standard errors clustered by cow ID. The ≥5% cervical PMN threshold is operational and does not establish a diagnosis of cervicitis. The data represent reproductive examinations; cows with repeat examinations were not treated as unique animals in descriptive counts.
